# Multimodal imaging for paracentral acute maculopathy; the diagnostic role of en face OCT

**DOI:** 10.1186/s40942-021-00283-y

**Published:** 2021-02-16

**Authors:** Hamid Riazi-Esfahani, Elias Khalili Pour, Kaveh Fadakar, Nazanin Ebrahimiadib, Fariba Ghassemi, Ramin Nourinia, Hassan Khojasteh, Behnoosh Attarian, Hooshang Faghihi, Hamid Ahmadieh

**Affiliations:** 1 Retina Service, Farabi Eye Hospital, Tehran University of Medical Sciences, Tehran, Iran; 2 Ophthalmic Research Center, Research Institute for Ophthalmology and Vision Science, Shahid Beheshti University of Medical Sciences, Tehran, Iran

**Keywords:** En face OCT, Multimodal imaging, Paracentral acute middle maculopathy

## Abstract

**Background:**

To describe the features of multimodal imaging and the diagnostic role of en face OCT in the paracentral acute middle maculopathy (PAMM) spectrum.

**Methods:**

In this observational case series, 5 eyes of 5 patients with acute PAMM were identified. Demographic characteristics as well as data regarding the underlying disease, presenting visual acuity (VA) and ophthalmic examination results were recorded. All patients underwent multimodal imaging within 3 days after symptom onset.

**Results:**

The mean age of patients was 52.2 (range, 33–67) years. Systemic comorbidities including diabetes mellitus and hypertension were identified in two patients. Except for one patient diagnosed with isolated PAMM, other patients had signs of retinal vascular disease such as a cilioretinal artery or branch retinal artery obstruction, non-ischemic central retinal vein occlusion, or a combination of these vascular disorders. The central vision was preserved in two cases; however, the remaining cases presented with profound VA reduction. Different patterns of PAMM including arterial, globular, and fern-like were observed in en face OCT at deep capillary plexus (DCP) level. En face OCT images could precisely delineate the margin of the PAMM area. Optical coherence tomography angiography (OCTA) showed decreased vascular density in DCP. Unresolved projection artifact by conventional OCTA software was observed in DCP and choriocapillaris slabs in all cases.

**Conclusion:**

En face structural OCT in PAMM can delineate the area of ischemia and the degree of foveal involvement. Unresolved projection artifact by conventional OCTA software in the PAMM area can be seen in DCP and choriocapillaris layers.

## Background

Paracentral acute middle maculopathy (PAMM) is the result of an ischemic insult to the deep or intermediate capillary plexus, affecting the middle retinal layers [1, 2]. It appears in cross-sectional optical coherence tomography (OCT) B-scans as a hyper-reflective band involving the inner nuclear layer (INL) [3–5]. PAMM may occur in isolation or secondary to various retinal disorders such as retinal arterial or venous occlusions and retinal vasculitis, or it may happen as a consequence of arterial hypoperfusion such as globe compression, migraine, or excessive caffeine consumption [1, 6, 7].

OCT angiography (OCTA) has provided better insight into the nature of the retinal vascular pathologies including diabetic retinopathy (DR), central retinal vein occlusion (CRVO), and arterial hypoperfusion [8–12]. OCTA delivers depth-resolved retinal vascular structure images, making it possible to differentiate capillary plexuses at superficial and deep layers of the retina; a phenomenon in which fluorescein angiography (FA) as the conventional standard for evaluation of retinal vascular diseases fails to identify due to the superimposition of the superficial capillary plexus over the deep capillary plexus as well as being masked by leakage and hemorrhage [7, 7]. En face OCT also known as C-scan OCT is an advanced processing application of OCT images created by participating the information from whole A-scans into a picture of the retinal surface in the desired slab, allows frontal portions of retinal layers to be viewed precisely. The en face images are equal to the depth-resolved retinal fundus images with additional focus on specific sites within the region of interest [9, 10].

In this case series, we are going to describe various presentations of PAMM using multimodal imaging. We emphasize the utility of en face OCT to reveal the pattern and extension of the ischemic area.

## Methods

This study was an observational case series of 5 patients with PAMM presenting to the retina clinics at Farabi Eye Hospital and Labbafinejad Medical Center, Tehran, Iran. Informed consent was obtained from all participants. This study adhered to the tenets of the Declaration of Helsinki and was approved by the ethics committees at both centers.

Patients with isolated PAMM or in association with arterial or venous occlusion were included in this study. The diagnosis was made through the ophthalmic exam and ancillary tests including SD-OCT, en face OCT, and OCTA. A thorough investigation including medical history, presenting best-corrected visual acuity (BCVA), slit-lamp biomicroscopic findings, and results of fundus examination were recorded.

Following imaging modalities were requested for each patient: color fundus photography (TRC.NW8F, Topcon, Japan), SD-OCT (Spectralis, Heidelberg Engineering, Heidelberg, Germany) and en face OCT and OCTA (Optovue, Fermont, California, USA); Topcon DRI OCT Triton SS-OCTA (Topcon, Tokyo, Japan) was also captured in one case. SD-OCT imaging was performed to capture relevant images with an axial resolution of 7 µm. Both eyes were scanned with a raster protocol of 20° × 2 0° quadrangular area centered at the fovea. Different patterns of PAMM including arteriolar (along the major retinal artery), globular (around distal capillaries), and fern-like (along the retinal veins) were investigated using en face structural OCT reconstruction with manually corrected segmentation at the INL level [3–5].

A 3*3 and 6*6 mm OCTA images, composed of 304 A-scans in each B-scan and centered at the fovea were captured. AngioVue uses a split-spectrum amplitude-decorrelation angiography (SSADA) algorithm to extract the data. The automatic segmentation provided by the device module was used to visualize the level of vascular pathology. Superficial capillary plexus (SCP) was segmented from 3 µm beneath the internal limiting membrane to 15 µm beneath the outer border of the inner plexiform layer (IPL). DCP was segmented from this point down to 9 μm below the outer plexiform layer (OPL). Whenever the segmentation lines were not correctly aligned according to the parameters defined above, the automatic segmentation was corrected manually by an expert (KF), using the ‘Edit Band/Propagation’ tool on the device software. The segmentation correction began from a single most central B scan and the propagation tool was used to automatically spread the correction to other adjacent B scans [11]. En face OCT and OCTA images in superficial and deep retinal capillary plexuses of each case were extracted.

## Results

Five eyes of five patients with the diagnosis of PAMM were included. Three (60%) were female. The mean age of patients was 52.2 (range, 33–67) years. Underlying co-morbidities including diabetes mellitus (DM) and systemic hypertension existed in two patients. Four patients had associated vascular occlusion or hypoperfusion in the territory of the cilioretinal artery, branch retinal artery, or central retinal vein. All images were captured within 3 days of the onset of symptoms. BCVA was not affected in two patients and the remaining cases presented with a profound decrease in visual acuity. Different patterns of PAMM including arterial, globular, and fern-like were observed in the en face OCT image at the level of DCP in all cases; en face OCT images could precisely delineate the margin of the PAMM area. One case at the level of a cotton wool spot showed severe inner retinal infarction. Unresolved projection artifact of superficial retinal vasculature on DCP and choriocapillaris slabs by built-in software of OCTA device was observed in all patients.

### Case presentations

#### Case 1: PAMM with cilioretinal artery occlusion (CILRAO)

**Fig. 1:**
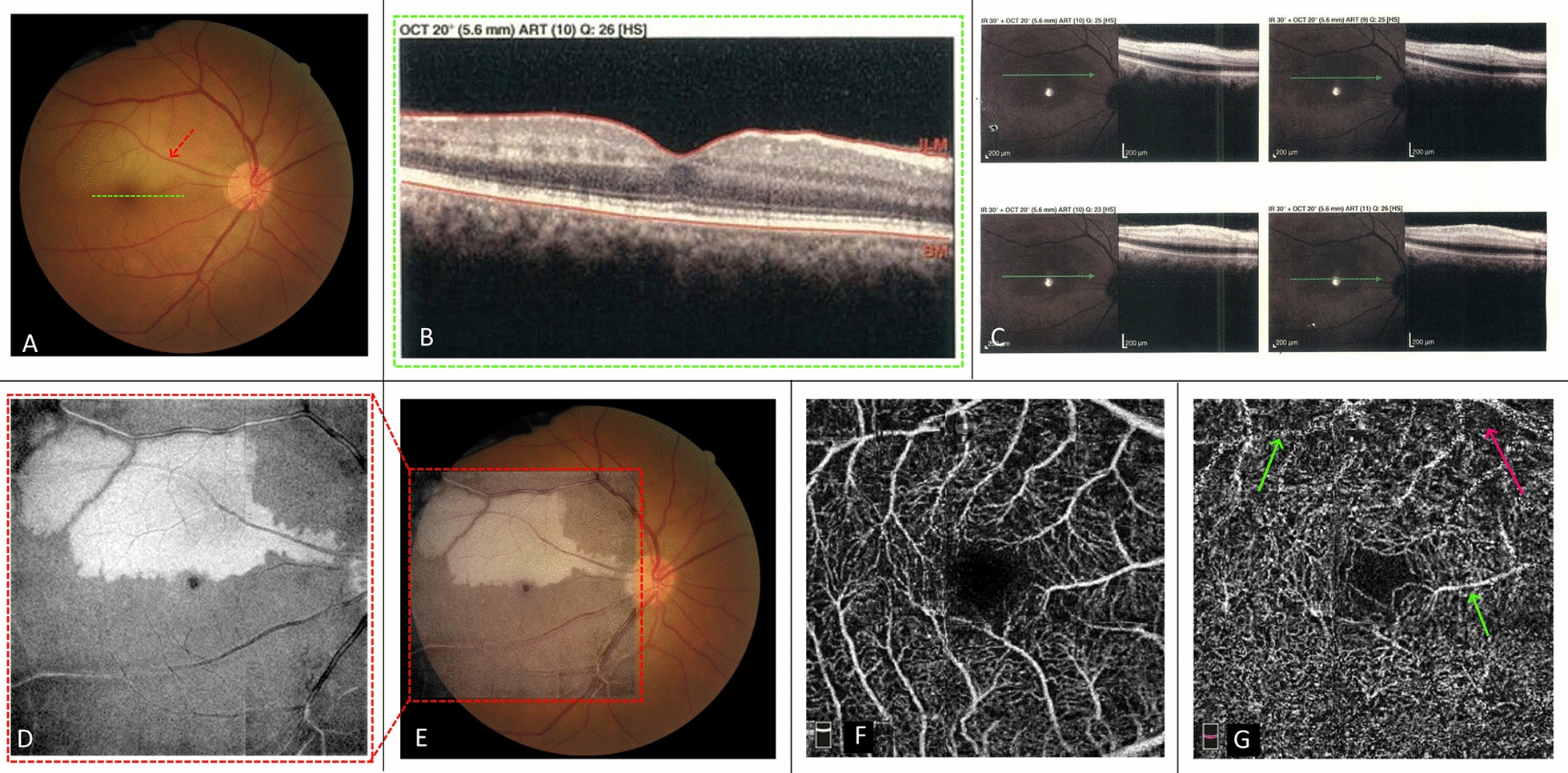
Fundus photograph of the right eye shows whitening and retinal edema around the cilioretinal artery (red arrow) (**a**). The foveal B-scan SD-OCT cut (corresponding to the green dashed line in color fundus photograph) shows temporal hyper-reflective bands involving INL with extension to IPL, consistent with the diagnosis of PAMM in the lower margin of the ischemic zone (**b**). B-scan cuts corresponding to the ischemic areas around the cilioretinal artery in the superior part of the fovea (**c**). In the central ischemic areas around the hypoperfused cilioretinal artery, SD-OCT shows involvement of both inner and middle retinal layers. The en face OCT image from DCP slab and its overlay on the fundus image is shown in **d**, **e**, respectively. As have been shown precisely by the en face OCT image, hyperreflectivity induced by ischemia around the involved cilioretinal artery is not seen in the foveal region. **f**, **g** show the superficial and deep capillary plexus slabs of the OCTA, respectively. DCP slab shows slight decreased vascular density in the superior half of the 3 × 3 image (red arrow) especially in areas closer to the cilioretinal artery and unresolved projection artifact of superficial vessels over deep layer in the superior part of the image (green arrows)

A 42-year-old man presented with the complaint of a paracentral scotoma in his right eye in the past 2 days upon awakening in the morning. He was a non-smoker, with well-controlled DM and systemic hypertension. BCVA was 20/20 in both eyes. Examination of the anterior segment was unremarkable. The fundus exam of the right eye revealed a well-defined yellow-white lesion in the triangular distribution pointing to the optic nerve along with the territory of the cilioretinal artery (Fig. 1a). SD-OCT demonstrated a local hyper-reflectivity as well as thickening of the inner and middle retinal layers within the involved cilioretinal artery territory. Scanning the fovea, parallel to the borders of the lesion, revealed a hyper-reflective band involving INL with extension to the inner plexiform layer (IPL), consistent with PAMM associated with cilioretinal artery insufficiency (Fig. 1b, c). The en face OCT image showed a well-demarcated hyperreflective area around the involved cilioretinal artery but the foveal center was spared explaining the good central vision in this patient (Fig. 1d, e). OCTA at the level of DCP showed slight capillary drop-out with visibility of the superficial retinal vessels due to projection artifact. Dropout was not significant at the site of PAMM corresponding to the border of the ischemic zone, but the decrease in vascular density was more pronounced in the central ischemic areas around the hypoperfused cilioretinal artery (Fig. 1f, g). Examination and imaging of the left eye were within normal limits. After 9 months follow-up, the 20/20 vision was preserved but he continued to complain of the paracentral scotoma in his right eye.

#### Case 2: PAMM with CILRAO

**Fig. 2:**
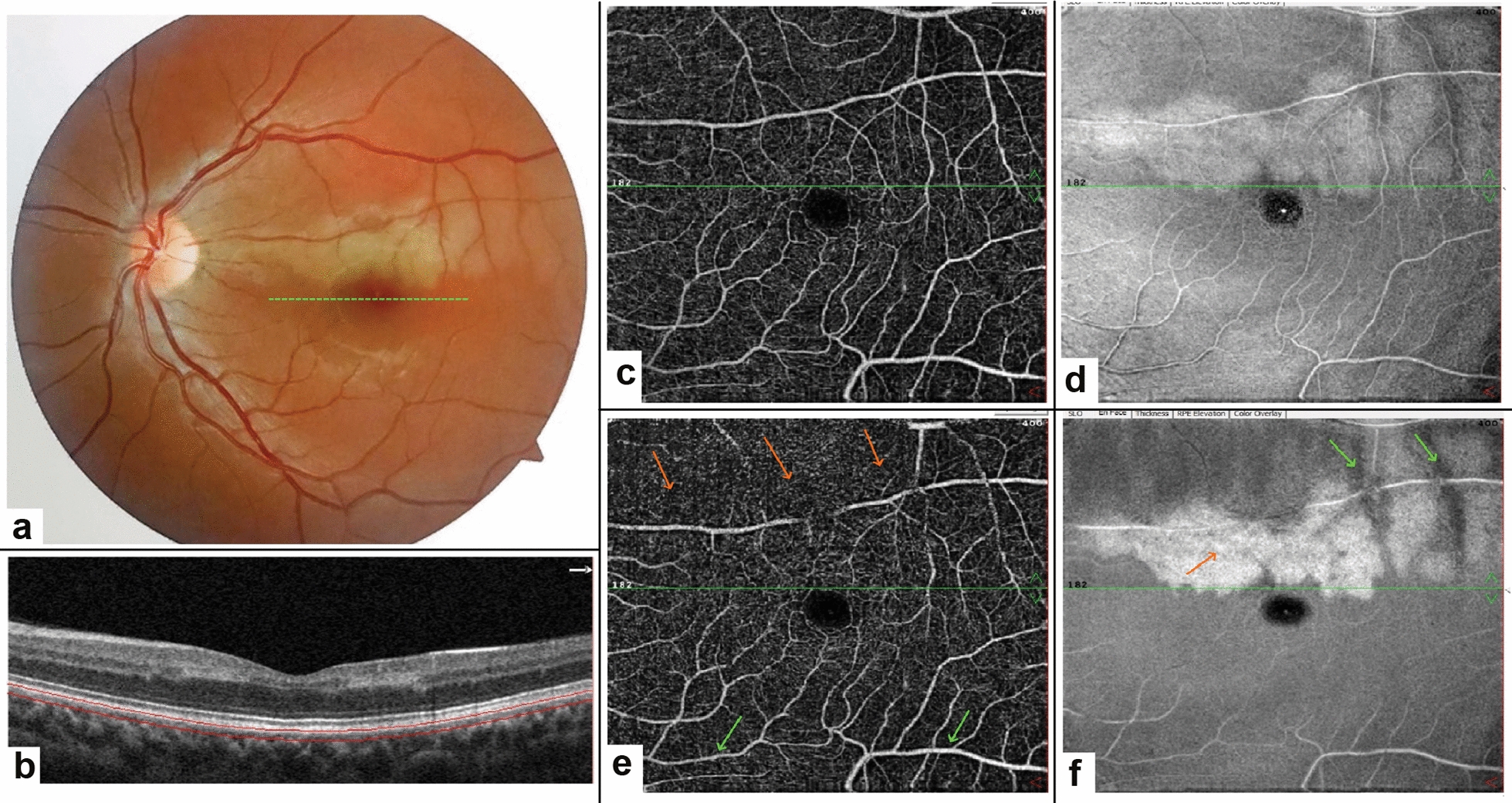
Fundus photography of the left eye shows whitening and edema around the hypoperfused cilioretinal artery (**a**). The OCT B-scan image of the patient is shown at the level of the green dashed line in the foveal area (**b**). The hyperreflective band is seen on both the temporal and nasal sides of the fovea in the INL. The OCTA and en face OCT images in the SCP have been shown in **c**, **d** respectively. Vascular density in SCP appears normal and en face OCT shows hyperreflectivity around the involved cilioretinal artery. The OCTA in the DCP **e** shows decreased vascular density in the area around the ciliary artery (orange arrows). In addition to the upper half of the 6 × 6 OCTA image, the projection artifact is well seen in the lower part of the fovea (green arrows) (**e**). The en face OCT image from the DCP area well indicates the extent and border of the hypoperfusion area as a hyperreflective region (**f**). En face OCT image at the level of DCP demonstrated a confluent globular hyperreflective area (orange arrow) in the territory of the cilioretinal artery. A perivenular hyper-reflectivity with periarterial sparing was evident at the superior border of the main ischemic area (green arrows)

A 33-year-old woman complaining of a sudden onset paracentral scotoma in her left eye was referred to the clinic. Her visual acuity was 20/20 in both eyes. Fundus exam of the left eye revealed pallor in the distribution of the cilioretinal artery, sparing the fovea (Fig. 2a). SD-OCT showed hyper-reflective bands at the INL extending from IPL to OPL without the involvement of the superficial layers indicating PAMM (Fig. 2b). FA demonstrated a filling delay of the cilioretinal artery (not included in Fig. 2). OCTA of the left eye showed reduced DCP vascular density surrounding the area of ischemia. En face OCT image at the level of DCP demonstrated a confluent globular hyperreflective area in the territory of the cilioretinal artery. A perivenular hyper-reflectivity with periarterial sparing was evident at the superior border of the main ischemic area (Fig. 2c-f). After about 1 year, BCVA was stable but the paracentral scotoma was persistent in the left eye.

#### Case 3: PAMM with branch retinal artery occlusion (BRAO)

**Fig. 3:**
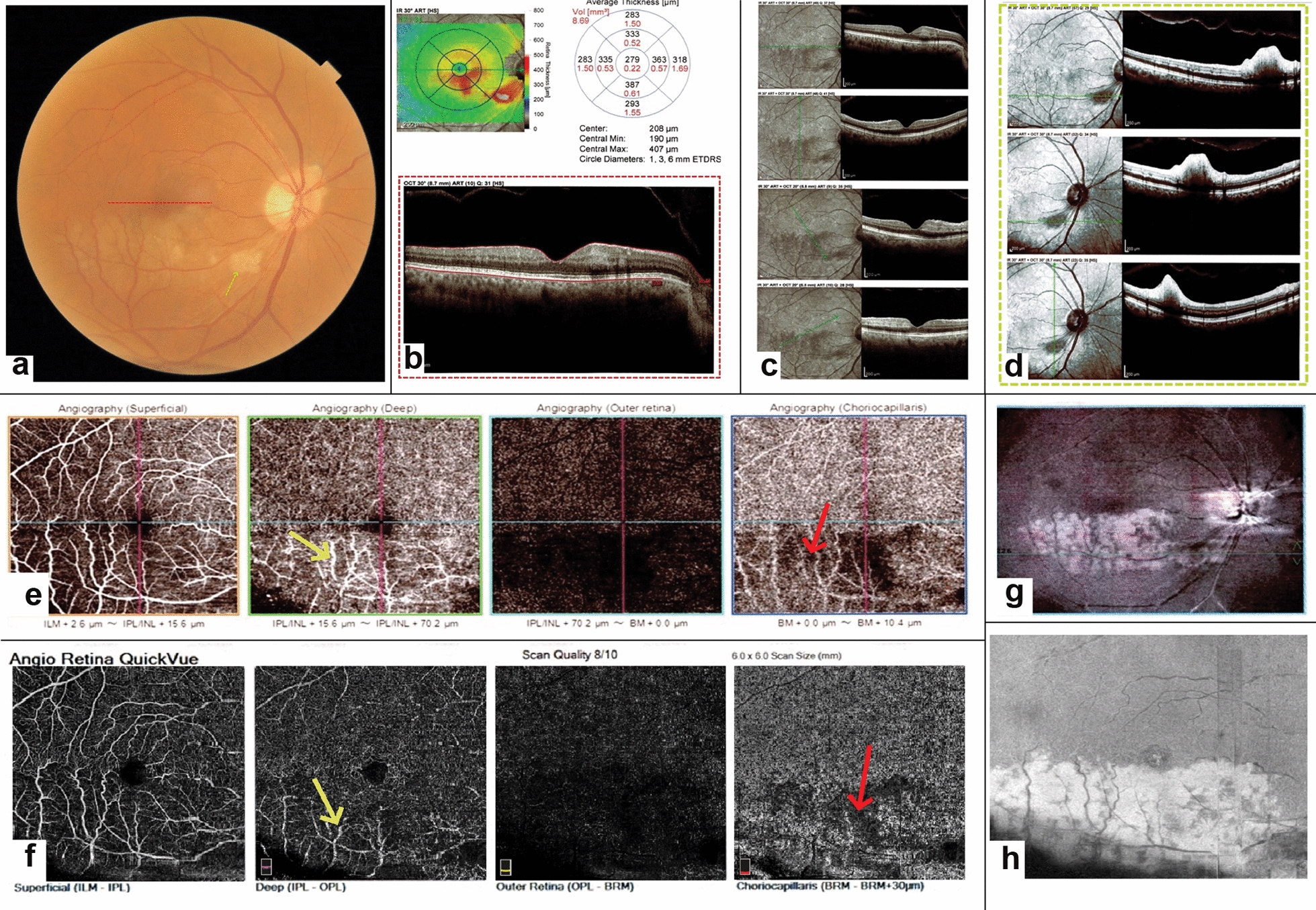
Fundus photography of the right eye shows a localized area of retinal whitening and edema corresponding to a cotton-wool spot (green-dashed arrow) and extension of this whitening and retinal edema around the inferotemporal branch retinal artery (**a**). The foveal B-scan SD-OCT cut (corresponding to the intersecting red line in **a**) is shown in **b**. As it can be seen, temporal and more pronounced nasal hyper-reflective bands have involved INL with extension to IPL, consistent with PAMM in the upper margin of the ischemic zone. Vertical and oblique B-scan cuts corresponding to ischemic areas around the involved inferior branch retinal artery are shown in **c**. The horizontal, vertical, and oblique B-scan images of the cotton-wool spot area are shown in **d**. **e**, **f** show the superficial and deep capillary plexus, outer retinal and choriocapillaris slabs of the OCTA by two different devices (**e** Topcon DRI OCT Triton SS-OCTA (Topcon, Tokyo, Japan) and **f** Optovue RTVue XR Avanti (Optovue, Inc, Fremont, CA)) respectively. DCP slab shows decreased vascular density in the inferior half of the 6 × 6 image especially in areas closer to the branch retinal artery and unresolved projection artifact of superficial vessels over the deep layer in the inferior part of the image (yellow arrows). Interestingly, the border of the area of flow voids could be better delineated at the choriocapillaris slab (red arrows). Corresponding en face OCT image from the DCP area is shown in **g**, **h**. En face OCT at the level of DCP clearly showed a sharp and well-demarcated fern-like whitening as a hyper-reflective area more prominent around the venules around the site of the involved retinal artery

A 56-year-old man came to the clinic with the complaint of decreased visual acuity in his right eye from the day before. BCVA was counting fingers at 2 m. The fundus exam showed an area of white plaque at the bifurcation of the retinal artery accompanied by a cotton-wool spot (CWS) in the distribution of the obstructed inferotemporal branch of the central retinal artery (Fig. 3a). FA showed an area of blockage as a result of CWS (figure not included). SD-OCT revealed a hyper-reflective band at the middle layers sparing more superficial layers of the retina except for the CWS region that showed involvement of both superficial and middle retinal layers (Fig. 3b–d). Evaluation of the patient’s multimodal imagings indicated branch retinal artery hypoperfusion which presented as PAMM and CWS. SD-OCT of the other eye was unremarkable. OCTA at the level of DCP showed scattered areas of vascular flow voids and projection of superficial retinal vessels over DCP (Fig. 3e, f). Interestingly, the border of the area of flow voids could be better delineated at the choriocapillaris slab. En face OCT at the level of DCP clearly showed a fern-like whitening as a hyper-reflective area more prominent around the venules around the site of the involved retinal artery (Fig. 3g, h). SD-OCT and OCTA of the other eye were normal. On the 4th day of follow-up, mild visual acuity improvement was noticed. After about 1 year, the CWS was resolved but the vision persisted to be 20/400.

#### Case 4: Isolated *PAMM*

**Fig. 4:**
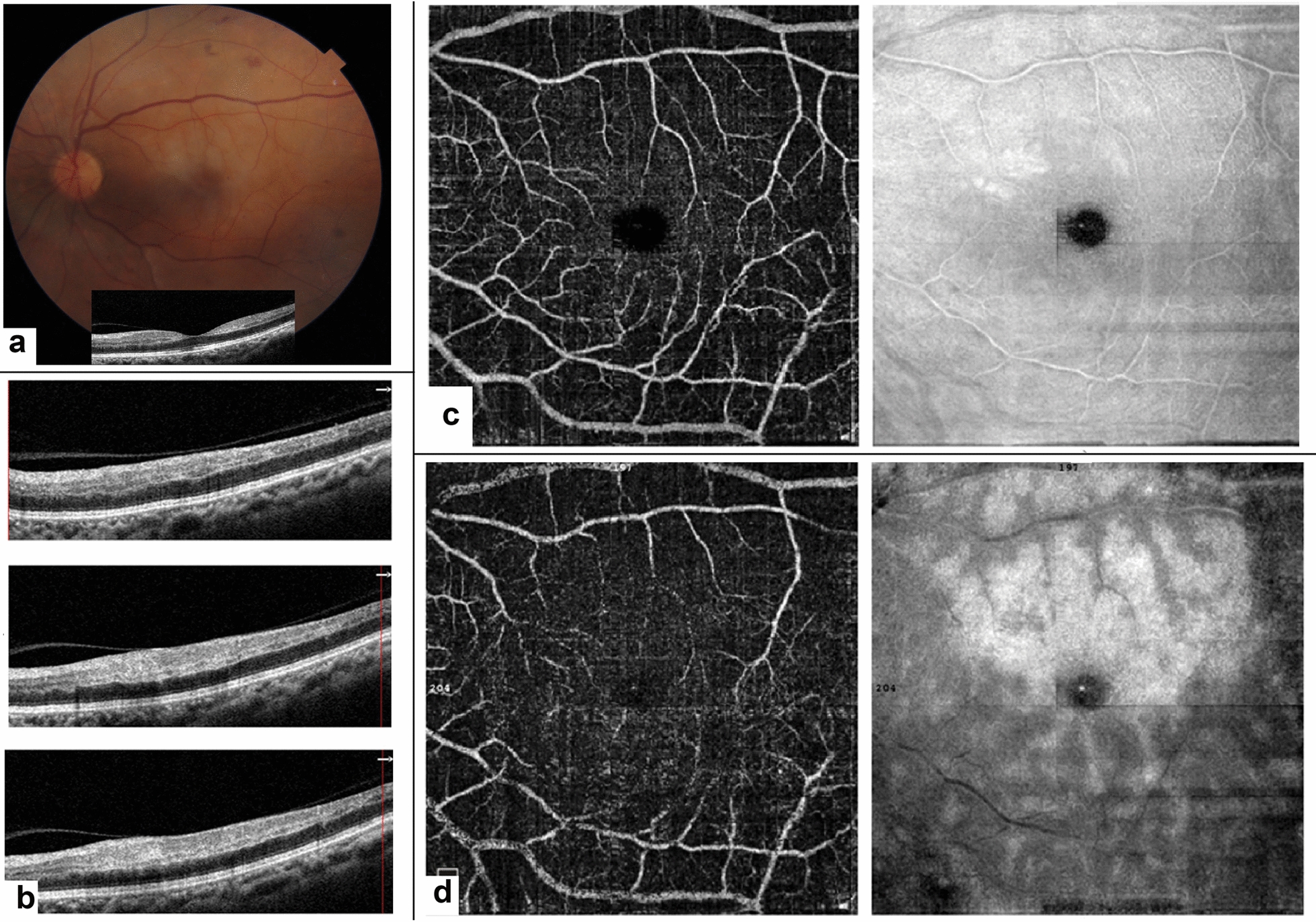
Color fundus photography of the left eye shows whitening and edema at the superior region of the macula (**a**). The foveal B-scan SD-OCT cut is shown below the fundus photography. The temporal side of fovea shows a hyperreflective band in the INL consistent with PAMM diagnosis. The OCT horizontal B-scan image of the patient at three different levels above the fovea shows the INL hyperreflective band (**b**). The OCTA image in the SCP and DCP besides the corresponding en face-OCT images at these levels have been shown in **c**, **d**. DCP image (4-D) shows a decrease in vascular density around the fovea. Also, the projection artifact is nicely seen in the upper and lower parts of the fovea. The en face OCT image at the level of DCP well designates the extent and border of the hypoperfusion area as a hyperreflective fern-like region and also depicted involvement of the fovea, explaining the reduced central vision in this patient

A 63-year-old woman with DM complained of sudden visual loss and altitudinal field defect in her left eye. BCVA was 20/20 (OD) and counting fingers at 2 m (OS). Fundus examination of the left eye showed pallor and edema in the superior portion of the macula. SD-OCT showed a hyper-reflective band in the temporal side of the fovea in the middle retinal layers consistent with PAMM (Fig. 4a). The OCT horizontal B-scan image of the patient at three different levels above the fovea shows the INL hyperreflective band (Fig. 4b).OCTA showed subtle areas of flow void at the superior part of the macula, especially in the DCP slab. En face OCT at the level of DCP demonstrated the extension of the lesion as a hyperreflective area, most prominently around the venules with relative sparing of the arterioles, making a fern-like appearance. En face OCT image at the level of DCP depicted the involvement of the fovea, explaining the reduced central vision in this patient’s left eye (Fig. 4c, d). The BCVA was still counting fingers at 2 m after 8 months.

#### Case 5: PAMM associated with non-ischemic CRVO and CILRAO

**Fig. 5:**
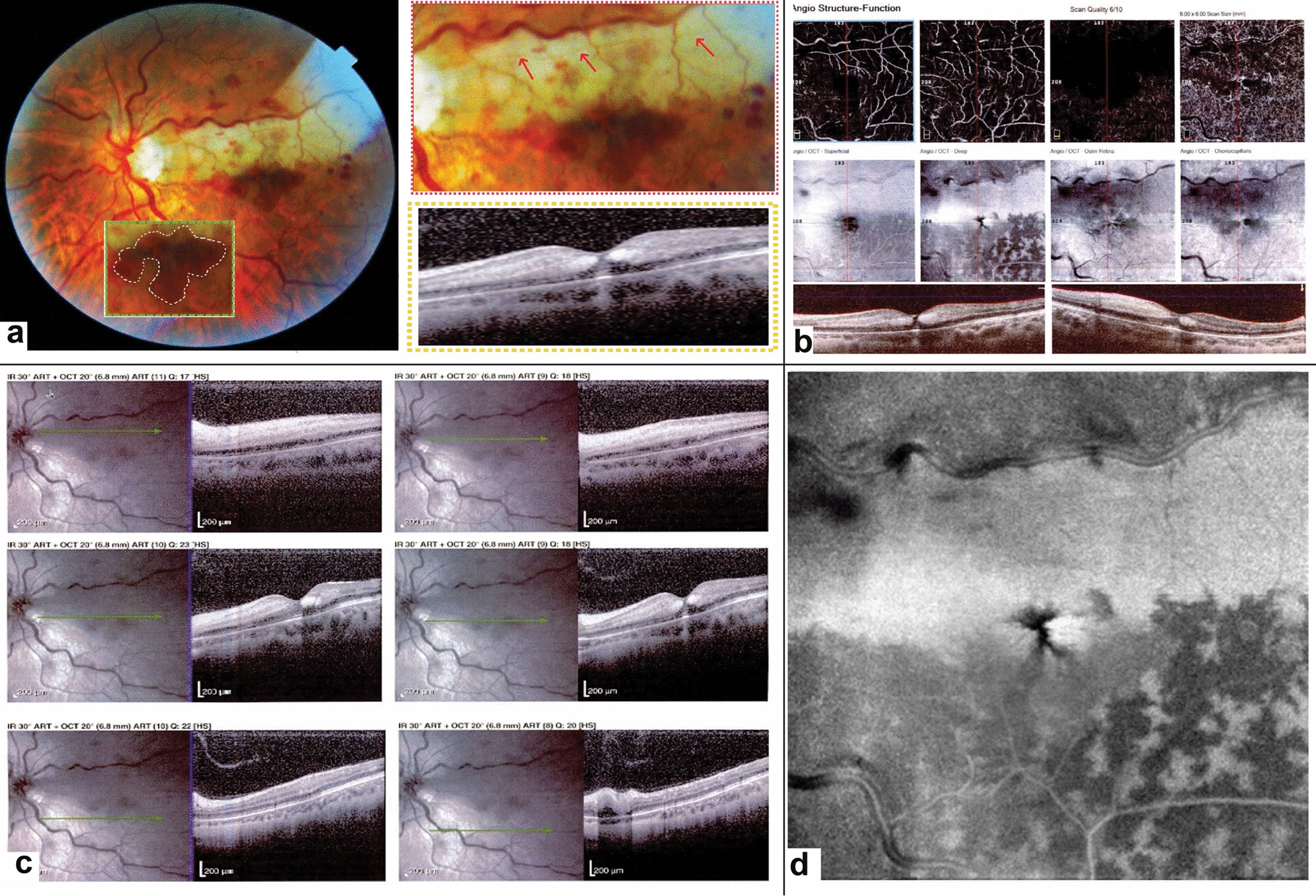
Fundus photography of the left eye three days after the onset of decreased visual acuity shows engorged and tortuous retinal veins with intraretinal hemorrhages consistent with the diagnosis of CRVO (**a**). There was an area of whitening in the territory of the cilioretinal artery compatible with the diagnosis of cilioretinal artery occlusion (magnified view: red dashed rectangle). The cilioretinal artery path is marked with red arrows. The foveal B-scan SD-OCT cut is shown in a yellow dashed rectangle. Hemorrhages with a radial pattern were encircling the fovea inferiorly. Borders of the hemorrhage have been shown in white dashed lines in the green dashed rectangle. The OCTA image of the left eye shows a decrease in vascular density in the SCP and DCP (**b**). The projection artifact of superficial arteries is well seen in the deep layer, which the software used in the Optovue device for omitting the projection artifact could not remove it. SD-OCT showed a slight increase in retinal thickness. A hyperreflective band in the area of CLRAO was observed in SD-OCT involving both superficial and middle retinal layers, while the lower border of the lesion appeared hyper-reflective solely in the middle retinal layers compatible with PAMM (**c**). En face OCT segmented at the level of DCP (**d**) shows the hyperreflective area in the distribution of the cilioretinal artery as well as base-out wedge shape hyperreflective lesions in the fovea corresponding to the oval-shaped hemorrhages. Fern-like perivenular hyperreflective lesions were also evident in the infratemporal part of the fovea in the region solely affected by venous occlusion

A 67-year-old woman was referred to our clinic with reduced vision to counting fingers at 1 m in her left eye in the past 3 days. Fundus exam revealed engorged and tortuous retinal veins associated with intra-retinal hemorrhages consistent with the diagnosis of CRVO (Fig. 5a). There was an area of whitening in the territory of the cilioretinal artery as a consequence of arterial hypoperfusion due to venous congestion. In addition, patches of retinal hemorrhage with a radial pattern were encircling the fovea inferiorly. SD-OCT showed increased retinal thickness and intra-retinal microcystic spaces (Fig. 5b). The area of cilioretinal hypoperfusion appeared to be hyper-reflective in SD-OCT; involving both the superficial and middle retinal layers while the lower border of the lesion appeared hyper-reflective solely in the middle layer. In addition, an oval-shaped hyper-reflective area bordering foveal center was observed in the Henle layer corresponding to the radially oriented hemorrhages. OCTA demonstrated complete loss of flow in the ischemic area (Fig. 5b, c). En face OCT image showed hyper-reflectivity in the distribution of the cilioretinal artery which involved the fovea. The area inferotemporal to the fovea showed perivenular hyper-reflectivity in en face OCT with a fern-like appearance (Fig. 5d). After about 6 months, the Henle layer hemorrhages were resolved and the patient mentioned a mild improvement in her vision. The BCVA reached just to 20/200 after 10 months. The SD-OCT showed decreased retinal thickness in the territory of the cilioretinal artery and disappearance of all intraretinal microcystic spaces.

Summarized clinical and multimodal imaging aspects of all patients have been shown in Table 1.

**Table 1:** Summarized clinical and multimodal imaging aspects of patients

	Demographic	PAMM Association	Complaint	BCVA	OCT	OCTA	Projection artifact	Enface	Final BCVA
Patient 1	42-year-old man	Cilioretinal artery occlusion (CILRAO)	Paracentral scotoma	20/20	The borders of the lesion revealed a hyper-reflective band involving INL with extension to inner plexiform layer	Superficial and deep capillary drop-out	+	Hyper-reflectivity in the distribution of cilioretinal artery	20/20
Patient 2	33-year-old woman	Cilioretinal artery occlusion (CILRAO)	Paracentral scotoma	20/20	Hyper-reflective bands at the INL extended from inner plexiform to outer plexiform layers	Deep capillary drop-out	+	The confluent globular hyperreflective area in the territory of the cilioretinal artery	20/20
Patient 3	56-year-old man	Branch retinal artery occlusion (BRAO)	Sudden visual loss	CF 2 m	The hyper-reflective band at the middle layers sparing more superficial layers of the retina except for the CWS region that showed involvement of both superficial and middle retinal layers	Superficial and deep capillary drop-out	+	A fern-like whitening as a hyper-reflective area more prominent around the venules around the site of the involved retinal artery	20/400
Patient 4	63-year-old woman	Isolated PAMM	Sudden visual loss and altitudinal field defect	CF 2 m	Hyper-reflective band of the middle retinal layers	Deep capillary drop-out	+	Hyper-reflective area, most prominently around venules with relative sparing of arterioles, making a fern-like appearance	CF 2 m
Patient 5	67-year-old woman	Non-ischemic CRVO and CILRAO	Sudden visual loss	CF 1 m	The area of cilioretinal hypoperfusion appeared to be hyper-reflective in both superficial and middle retinal layers while the lower border of the lesion appeared hyper-reflective solely in the middle layer	Superficial and deep capillary drop-out	+	Hyper-reflectivity in the distribution of cilioretinal artery, inferotemporal to the fovea, showed perivenular hyper-reflectivity with a fern-like appearance	20/200

PAMM: Paracentral acute middle maculopathy, CRVO: central retinal vein occlusion, BRAO: Branch retinal artery occlusion, CILRAO: Cilioretinal artery occlusion, BCVA: best corrected visual acuity, OCT: Optical coherence tomography, CF: count finger, INL: Inner nuclear layer, CWS: cotton wool spot

## Discussion

In this case series, we presented a spectrum of PAMM associated with various clinical settings including CILRAO, BRAO, CRVO plus CILRAO, and an isolated PAMM. All patients were evaluated within 3 days of the onset of the ischemic event. We described the findings of multimodal imaging and identified en face OCT as the most useful modality in the diagnosis and determination of the extension of the lesion. Color fundus photograph and OCT helped us to establish the diagnosis of an ischemic insult. However, as illustrated in our series, assessment of the true extension of the lesion and the status of the fovea could be better made with the aid of structural en face OCT. This feature was especially well-illustrated in our fourth patient, who had a visual acuity of counting fingers in the involved eye. Based on the fundus photograph and OCT, the fovea seemed to be mildly involved; however structural en face OCT revealed the true severity of foveal involvement as the explanation for reduced central vision. Similarly, in our first patient with cilioretinal artery occlusion and PAMM whose central vision was unaffected, OCT could be misleading, since the hyper-reflectivity in the middle retinal layer was in approximation with the fovea. However, en face structural OCT demonstrated the foveal sparing in this patient, in concordance with the preserved central visual acuity. Similar findings could be appreciated in our second and fourth patients. After 8–10 months, the BCVA was not significantly changed compared to baseline in all cases, and the final visual acuity correlated with the status of the fovea based on the baseline en-face structural OCT findings. Previous studies showed a positive correlation between the subjective evaluation of scotoma by microperimetry and areas of ischemia revealed by multimodal imaging [7]. As an objective and non-invasive test, structural en face OCT image acquisition should be considered in PAMM to better delineate the extension of the lesion and predict the visual prognosis.

Another exclusive finding in our fifth patient presenting with non-ischemic CRVO and cilioretinal hypoperfusion associated with PAMM was the presence of radial hemorrhages surrounding the fovea. These hemorrhagic spots were located in the Henle layer (OPL) and the INL which appeared hyperreflective in OCT. Sarraf et al. first described this finding in patients with macular telangiectasia type 2 (MacTel2) [12]. They presented three patients with MacTel2 who had a characteristic radial hemorrhage localized in the OPL of Henle using structural en face OCT. As there was no evidence of macular neovascularization, they hypothesized that the hemorrhage in OPL developed as a consequence of DCP microvascular events secondary to the abnormality of this layer. We observed a similar finding in our patient with non-ischemic CRVO (case 5). In patients with CRVO, increased venous pressure due to occlusion of the central retinal vein causes congestion and increased pressure in DCP which has a vortex-like pattern based on OCTA findings [13, 14]. Considering the Henle fiber layer is located in the outer border of the outer plexiform layer, hemorrhage from DCP can appear in this layer with a petaloid or radial appearence [12, 15].

Although quantitative analysis of DCP vascular density showed a significant reduction in areas impacted by PAMM in comparison with the non-ischemic areas, these alterations were difficult to be detected without image processing or enhancements and might be overlooked [4]. Also, an increase in flow void areas with time has been proposed by Nemiroff et al. They demonstrated a nonsignificant decrease in vascular density of DCP in acute stages of PAMM while this turned to be significant with chronicity [4]. Similarly, in our first case, we did observe a very slight visible vascular dropout in the region of the PAMM lesion.

It is noteworthy that artifacts from the projection of superficial vasculature on middle retina slab were seen in all of our cases in en face OCTA, despite manually corrected segmentation and centration. The extension of this artifact was relevant to the area of hyper-reflectivity in en face OCT and it was more prominent in more profound ischemic events. It has already been shown that structures with high OCT reflectance such as hard exudates appear to amplify the signal from motion or projection artifact [16]. On the other hand, this projection artifact can be explained by the RPE-like hyperreflective nature of front involved layers during the PAMM which is not supposed to be eliminated by software. This projection artifact can also be appreciated in the choriocapillaris slab.

Interestingly, the area of ischemia can be delineated in the choriocapillaris slab whereas this is not defined in the area of ischemia itself (DCP). This phenomenon was observed in case 2 (BRAO) and case 5 (CRVO + CILRAO) which seemed to have more profound ischemia. Projection artifact might be the result of increased reflectiveness due to intracellular edema. Better delineation of the ischemic area in en face OCTA choriocapillaris slab could be the result of masking artifact caused by increased reflectivity of the upper layers which inhibited signal transmission from the lower segments [17].

Our PAMM cases demonstrated a spectrum of perivenular to globular and arteriolar involvement in en face OCT images. This fern-like (perivenular) pattern in en face OCT is pertinent to areas farther from the main ischemic event, where it is of less severity, and shows hyperreflectivity only in the middle retinal layers in B-scan OCT cuts, while in regions suffering from the most intense anoxic condition (i.e. closer to the involved artery), hyperreflectivity of both middle and inner retinal layers in B-scan OCT with a globular pattern in en face OCT is observed. Recent studies revealed that micro vortex-like structures in DCP were responsible for venous drainage of middle retinal layers and behaved as a watershed zone area [18–20]. Meticulous scrutiny of en face OCT and OCTA imaging has demonstrated that PAMM, whether isolated or not, heralds an ischemic cascade, which begins as a fern-like configuration with a propensity to venular pole at the level of DCP and progresses laterally to the center of ischemia, producing globular pattern. In more severe forms of ischemia such as arterial occlusion, an anterior extension might occur which leads to ischemia of the inner retinal layers [20]. The pattern of the extension indicates a serial organization of retinal capillary architecture, emphasizing the perivenular area at the DCP as the most sensitive segment to ischemic events. Additionally, it has been postulated that cell survival in the ischemic area is not only dependent on microcirculation but also on the oxygen tension at the nearest vessel. Therefore, cells in close approximation to larger arterioles may survive better in the anoxic conditions than those that are closer to the venular system [21].

Our study has some limitations including the small sample size with scarce longitudinal follow up data. Further prospective clinical studies with a larger sample size would be required to confirm the clinical significance of these findings.

In summary, in this series of 5 patients with acute PAMM, we showed that en face structural OCT is informative in delineating the area of ischemia and evaluating the foveal involvement and justification of reduced visual acuity, more than OCT and OCTA. In addition, unresolved projection artifact by conventional OCTA software was observed in the DCP and choriocapillaris slabs in our cases with both spectral-domain and swept-source OCTA. However, the PAMM involved region was more visible in the choriocapillaris slab of OCTA.

## Data Availability

The datasets used in the current study are available upon reasonable request.

## Abbreviations

PAMM: Paracentral Acute Middle Maculopathy
VA: Visual Acuity
OCT: Optical Coherence Tomography
SD-OCT: Spectoralis Domain- Optical Coherence Tomography
FA: Fluorescein Angiography
OCTA: Optical Coherence Tomography Angiography
SCP: Superficial Capillary
DCP: Deep Capillary Plexus
DM: Diabetes Mellitus
DR: Diabetic Retinopathy
CRVO: Central Retinal Vein Occlusion
BRAO: Branch Retinal Artery Occlusion
CILRAO: Cilioretinal Artery Occlusion
BCVA: Best Corrected Visual Acuity
SCP: Superficial Capillary Plexus
DCP: Deep Capillary Plexus
FAZ: Foveal Avascular Zone
IPL: Inner Plexiform Layer
OPL: Outer Plexiform Layer
INL: Inner Nuclear Layer
CWS: Cotton-Wool Spot
MacTel2: Macular Telangiectasia Type 2
