# Correction: Multimodal imaging for paracentral acute maculopathy; the diagnostic role of en face OCT

**DOI:** 10.1186/s40942-024-00589-7

**Published:** 2024-10-07

**Authors:** Hamid Riazi-Esfahani, Elias Khalili Pour, Kaveh Fadakar, Nazanin Ebrahimiadib, Fariba Ghassemi, Ramin Nourinia, Hassan Khojasteh, Behnoosh Attarian, Hooshang Faghihi, Hamid Ahmadieh

**Affiliations:** 1 Retina Service, Farabi Eye Hospital, Tehran University of Medical Sciences, Tehran, Iran; 2 Ophthalmic Research Center, Research Institute for Ophthal mology and Vision Science, Shahid Beheshti University of Medical Sciences, Tehran, Iran

**Correction: Int J Retin Vitr 7**,** 13 (2021)**

10.1186/s40942-021-00283-y

Following publication of the original article [1], it was reported that reference 15 was incorrectly published. The correct reference 15 is:

Baumal, CR., Sarraf, D., Bryant, T., et al. Henle fibre layer haemorrhage: clinical features and pathogenesis. *British Journal of Ophthalmology* 2021;**105**:374–380.

The original article [1] is corrected.
